# Predictors of suicide attempts among adolescents with suicidal ideations and a plan: Results from the National Survey on Drug use and Health (NSDUH)

**DOI:** 10.1371/journal.pone.0331261

**Published:** 2025-08-28

**Authors:** Michele Cherro, Hala Itani, Elias Ghossoub, Fadi Maalouf

**Affiliations:** 1 Department of Child and Adolescent Psychiatry, Massachusetts General Hospital, Boston, Massachusetts, United States of America; 2 Department of Psychiatry, American University of Beirut, Beirut, Lebanon; NYU Grossman School of Medicine: New York University School of Medicine, UNITED STATES OF AMERICA

## Abstract

**Purpose:**

Suicide remains an ongoing public health concern, especially among adolescents. While many studies investigated the transition from ideations to attempts, they did not specifically look at the factors underlying the transition from a suicide plan to a suicide attempt, creating a vast knowledge gap. In the following study, we aim to investigate predictors of suicide attempts among adolescents with a suicide plan using data from NSDUH.

**Methods:**

We used de-identified data from consecutive cross-sectional NSDUH surveys conducted between 2015 and 2018, including respondents aged 12–17 who reported suicidal ideations and a plan. We performed bivariate analyses and multivariate logistic regression analyses to identify significant predictors of suicide attempts in our population.

**Results:**

Our total sample included 3003 respondents (Population Size Estimate: 1,284,704.48). Among them, 1,780 reported suicide attempts (A) and 1,223 reported no suicide attempts (NA). The majority of adolescents in both groups were aged 15–17 years. More females were present in the (A) group compared to the (NA) group (p = 0.013). We found a positive association between antisocial behaviors and suicide attempts. Specifically, engaging in three or more antisocial behaviors significantly increased the odds of suicide attempts [adjusted odds ratio (OR) = 1.81; 95% CI = (1.11–2.96). Substance use and violent behaviors were also significantly associated with an increased suicide risk.

**Conclusion:**

We found significant correlations between suicide attempts and gender, substance use disorders and engaging in antisocial behaviors. These findings underscore the importance of addressing mental health issues, substance use, and antisocial behaviors in suicide prevention efforts for at-risk adolescents.

## Introduction

Suicide is one of the leading causes of death worldwide [1], especially among adolescents of ages 12–18 [2]. Despite extensive literature identifying risk and protective factors of suicidal behaviors [3], prevalence of completed suicides among the adolescent population continues to rise, making it an ongoing public health crisis [4].

Suicide terminology is often heterogeneous in the literature; however, one standard nomenclature suggests that suicidal behavior exists on a spectrum of severity [5]. It includes suicidal ideations (SI) which describe a variety of thoughts ranging from contemplation and wishes to preoccupation with death [6]. These thoughts can manifest with varying levels of seriousness and intensity including intent which refers to the severity of the patient’s wish to terminate his or her life as well as a plan which alludes to a specific method of carrying out the act [6]. Identifying the risk factors underlying the transition from one level to the other would be vital in suicide prevention efforts, especially due to the fact that only a third of those with suicidal ideations eventually go on to make an attempt [7].

Some of the risk factors that are common to suicidal ideations and attempts among adolescents include: previous suicide attempts [4], bullying [8], mental illness [9] and chronic medical non-psychiatric illnesses [10]. On the other hand, factors that differentiate youth with suicide attempt from those with suicidal ideations include exposure to suicidal behavior in family or friends, depressive disorders, anxiety disorders, and smoking among others [11]. In the National Youth Risk Behavior surveys, among youth with suicidal ideations, those who experienced rape, physical fights, and used heroin were more likely to attempt suicide [12].

Another risk factor for suicide is the presence of antisocial behavior during adolescence. In fact, a Swedish National Cohort Study published in 2011, identified that both females and males with convictions faced elevated suicide risks, with more severe delinquency independently associated with heightened risk of suicide in young adulthood [13].

Youth who die by suicide are more likely to have a parent or sibling with severe mental illness, a parent who died early or who died by suicide and to have a mental illness themselves as compared to age- and gender-matched controls in large national registries [14].

Most classic suicide risk factors in the literature are usually related to suicidal behaviors and do not always specify the predictors of the transition from ideations to a plan or from a plan to an attempt [15]. Suicide is often hypothesized to progress in seriousness from death wishes to suicidal ideations, then to planning and finally to an attempt with an exponentially increasing risk [16]. While many studies investigated the transition from ideations to attempts, they did not specifically look at the factors underlying the transition from a suicide plan to a suicide attempt, creating a vast knowledge gap [17]. In fact, planning is a very important factor to consider in conducting suicide risk assessments as it has been frequently associated with an increased risk of suicide [3]. Among individuals who have attempted suicide, 60% had prior suicide plans [7]. Additionally, an epidemiological study of adolescents conducted in Germany in 2019 found that most (85.4%) but not all individuals eventually transitioned from a suicide plan to an attempt, often within a year [18]. Hence, prompt and timely recognition of risk among adolescents who report suicide plans is of utmost importance.

Given the aforementioned knowledge gap, we aim to investigate the different predictors of suicide attempts among adolescents with a suicide plan, using data from the National Survey on Drug Use and Health (NSDUH).

## Methods

### Data source

We used the publicly available de-identified data of the National Survey on Drug Use and Health (NSDUH), pooling data from consecutive cross-sectional surveys from 2015 through 2018 [19]. IRB approval was waived since the data utilized came from a publicly available database that was de-identified. The NSDUH is a household survey conducted on a yearly basis and is representative of the United States non-institutionalized population aged 12 years and older in all fifty states and the District of Columbia [19]. Interviews are held face-to-face with computer-assisted interviewing, which provides participants with privacy and encourages them to answer honestly to questions about socio-demographic parameters, substance use and mental health [19]. Detailed methodology reports for each annual survey are available [19].

We used the data of respondents aged 12–17 years for our analyses. Respondents were asked in the survey to think about the period of time when their feelings and problems were the worst.

We included in our sample the respondents who answered yes to the following two questions with regards to that period of time: “*Did you think about killing yourself?”* and “*Did you make a plan to kill yourself?”.* Our total sample included 3003 respondents.

National Survey on Drug Use and Health (samhsa.gov)

### Measures

#### Dependent variable.

We designed our dependent categorical variable measuring attempted suicide based on the following question: “*Did you make a suicide attempt or try to kill yourself?”* (regarding the period of time when the respondents’ feelings and problems were the worst). The categories of our variable are:

- Attempters (A): a suicide attempt is reported.
- Non-Attempters (NA) no suicide attempt reported.

#### Variables *of* interest.

Our main variable of interest measures antisocial behaviors as reported by the respondents. We designed a composite variable (ASB) measuring antisocial behavior based on the following questions:

- *Not counting minor traffic violations, have you ever been arrested and booked for breaking the law?*
- *Not counting minor traffic violations, how many times during the past 12 months have you been arrested and booked for breaking a law?*
- *During the past 12 months, how many times have you gotten into a serious fight at school or work?*
- *During the past 12 months, how many times have you taken part in a fight where a group of your friends fought against another group?*
- *During the past 12 months, how many times have you carried a handgun?*
- *During the past 12 months, how many times have you sold illegal drugs?*
- *During the past 12 months, how many times have you stolen or tried to steal anything worth more than $50?*
- *During the past 12 months, how many times have you attacked someone with the intent to seriously hurt them?*
- *During the past 12 months, have you driven a vehicle while you were under the influence of a combination of alcohol and illegal drugs used together?*

The categories of our variable (ASB) are:

- None reported.
- One antisocial behavior reported.
- Two antisocial behaviors reported.
- Three or more antisocial behaviors reported.

Other variables of interest include age, sex, race/ethnicity, school attendance, household type, past-year employment, family income level, area of residence, lifetime major depressive episode, past-year major depressive episode, past-year tobacco use, past-year substance use disorder and past-year mental health treatment.

### Statistical analysis

We completed our statistical analyses using the Complex Samples module in the Statistical Package for Social Sciences (SPSS) version 21. We adjusted sample weights through computing a variable (We) according to the following formula available in the survey methodology books [19]:


$$(We)=\frac{Person-Level\;Analysis\;Weight}{Number\;of\;Years\;of\;Combined\;Data}=\frac{Final\;Person-Level\;Sample\;Weight}{4}$$

We conducted bivariate analyses for all variables with the dependent variable. We measured the associations using the adjusted F test, which is a variant of the Chi-Square test adjusted for complex samples. We set statistical significance at the alpha level of 5%. We also conducted Spearman’s correlation test among our main variables of interest to test for multicollinearity. We then conducted multivariate logistic regression analyses with the variables that were significant in bivariate analyses, and we adjusted the models to reach the most parsimonious model. Finally, we calculated the adjusted OR (aOR) and its corresponding 95% confidence interval (CI) for attempting suicide when having a positive self-report of engaging in antisocial behavior.

## Results

### Sample characteristics

Our total sample included 3,003 respondents aged 12–17. The sample is divided between 1780 suicide attempters (A) and 1223 non-attempters (NA). As shown in Table 1, there was a higher proportion of girls in the (A) group (80. 1% vs 75.7% in the NA group, p = 0.013) and adolescents in the (A) group came from families with lower income than those in the NA group (p = 0.035). The two groups did not differ on other demographic variables. Most adolescents in the (A) and (NA) groups were aged 15–17 years (69.3% in the A group and 66.1% in the NA group, p = 0.13) and were non-Hispanic white (54.6% in the A group and 58.8% in the NA group, p = 0.118).

**Table 1:** Population characteristics.

Characteristic	Suicide attempts (N = 1780; PSE = 769,353.655; P = 59.9%)	No suicide attempts (N = 1223; PSE = 515,351.822; P = 40.1%)	Adjusted F	p-value
	P (SE)	P (SE)		
**Age in years**			2.365	0.130
*12-14*	30.7 (1.4)	33.9 (1.8)		
*15-17*	69.3 (1.4)	66.1 (1.8)		
**Gender**			6.601	**0.013**
*Male*	19.9 (1.2)	25.3 (1.7)		
*Female*	80.1 (1.2)	74.7 (1.7)		
**Race**			2.018	0.118
*Non-Hispanic white*	54.6 (1.8)	58.8 (1.5)		
*Hispanic*	24.8 (1.5)	21.7 (1.3)		
*Non-Hispanic African American*	10.5 (0.9)	8.1 (0.8)		
*Other*	10.1 (1.0)	11.4 (1.1)		
**School**			0.195	0.661
	91.7 (0.9)	91.2 (1.0)		
**Employment**			0.488	0.488
	32.3 (1.9)	30.2 (2.2)		
**Family income**			3.041	**0.035**
*Less than $20,000*	15.5 (1.1)	12.4 (1.3)		
*$20,000 - $49,999*	31.5 (1.5)	27.4 (1.6)		
*$50,000 - $74,999*	15.6 (0.9)	17.4 (1.6)		
*$75,000 or more*	37.4 (1.1)	42.8 (2.3)		
**Parents in household**			2.043	0.135
*Both parents in household*	64.8 (1.5)	68.1 (1.8)		
*One parent in household*	28.4 (1.5)	27.2 (1.6)		
*No parents in household*	6.8 (0.7)	4.7 (0.8)		
**Population density**			0.129	0.861
Segment in a CBSA^*^ *with > 1 million*	50.3 (1.8)	51.5 (2.0)		
*Segment in a CBSA with <than 1 million*	43.7 (1.9)	42.7 (1.9)		
*Segment not in a CBSA*	6.0 (0.8)	5.8 (0.8)		
**Major depressive episode**			1.126	0.325
*MDE in the past year*	82.1 (1.2)	80.1 (1.4)		
*Lifetime MDE (excluding past year)*	17.1 (1.2)	18.5 (1.4)		
*No MDE*	0.7 (0.2)	1.4 (0.5)		
**Major depressive episode over the past year with severe role impairment**			5.66	**0.021**
	71.5 (1.3)	66.6 (1.5)		
**Spoke to a professional in the past year**			23.655	**<0.001**
	53.5 (1.1)	42.2 (1.9)		
**Received treatment/counseling or medications in the past year**			27.640	**<0.001**
	58.1 (1.1)	45.3 (2.0)		
**Tobacco use in the past year**			22.267	**<0.001**
	30.0 (1.2)	19.6 (1.6)		
**Alcohol use disorder in the past year**			30.739	**<0.001**
	11.5 (1.1)	5.0 (0.7)		
**Substance use disorder in the past year**			19.313	**<0.001**
	17.0 (1.3)	7.2 (0.9)		
**Cannabis use disorder in the past year**			35.528	**<0.001**
	11.0 (1.0)	5.1 (0.8)		
**Substance use disorder (without cannabis) in the past year**			48.992	**<0.001**
	9.8 (0.9)	2.8 (0.5)		
**Arrested**			6.803	**0.002**
*In the past year*	4.5 (0.6)	2.3 (0.6)		
*Not in the past year*	2.9 (0.7)	1.0 (0.3)		
*Never arrested*	92.6 (0.9)	96.7 (0.7)		
**On parole or probation in the past year**			4.397	**0.041**
	3.9 (0.5)	1.9 (0.5)		
**Moved over the past 12 months**			5.449	**0.024**
	35.9 (1.7)	29.3 (1.8)		
**Serious fight at school or work**			24.246	**<0.001**
*One or more times*	32.0 (1.4)	22.5 (1.5)		
**Fight in a group versus another group**			5.268	**0.026**
*One or more times*	18.9 (1.2)	15.1 (1.1)		
**Carried a handgun**			0.765	0.386
*One or more times*	5.3 (0.7)	4.3 (0.9)		
**Sold illegal drugs**			8.612	**0.005**
*One or more times*	7.5 (0.7)	4.0 (0.7)		
**Stole/tried to steal an item > $50**			6.543	**0.014**
*One or more times*	9.2 (0.8)	5.9 (0.8)		
**Attacked with intent to seriously harm**			6.251	**0.016**
*One or more times*	14.2 (1.2)	9.6 (1.1)		
**DUI** ^**^			8.845	**0.005**
	8.9 (0.7)	5.1 (0.9)		

* *CBSA: core-based statistical area*

** *DUI: Driving under the influence*

*Significant results in* ***Bold*** *(p < 0.05)*

Although around 80% of adolescents in both groups had a major depressive episode (MDE) in the past year (p > 0.05), adolescents in the (A) group were more likely to have had a depressive episode with severe impairment than those in the (NA) group (71.5% vs 66.6%, p = 0.021). Adolescents who attempted suicide were more likely to have received treatment/counseling or medications for MDE in the past year (58.1%) than those who did not attempt suicide (45.3%) (p < 0.001).

Of those who attempted suicide, 17% had a substance use disorder versus 7.2% among those who did not attempt suicide (p < 0.001). 11.5% of the (A) group had alcohol use disorder compared to 5.0% of the (NA) group (p < 0.001) while 30% of the A group used tobacco in the previous year compared to 19.6% in the NA group (p < 0.001). Adolescents in the (A) reported being more likely to have ever been arrested than those in the NA group (p < 0.002). 3.9% in (A) group and 1.9% in NA group were on parole or probation in the past year (p = 0.041). When looking at the different violence characteristics between those who attempted suicide and those who didn’t, it is observed that 32.0% of (A) group vs 22.5% in (NA) group had one serious fight or more at school or work (p < 0.001). Few adolescents in both groups had carried a handgun (5.3% in (A) group and 4.3% in (NA) group, p = 0.386), sold illegal drugs (7.5% in (A) group and 4.0% in (NA) group, p = 0.005) or stole/tried to steal an item worth> $50 (9.2% in (A) group and 5.9% in (NA) group, p = 0.014). Moreover, of those who attempted suicide, 14.2% had attacked with intent to seriously harm once or more, while of those who didn’t attempt suicide, 9.6% did. This difference was statistically significant (p = 0.016).

### Multinomial logistic regression (Table 2)

We did not find significant multicollinearity among our main variables of interest after running the Spearman’s correlation test. All categories of ASB were statistically significant in the unadjusted logistic regression model. When adjusting for age, sex, race/ethnicity and survey year, these results remained significant (Model 1). There is an incremental association with the number of reported ASB. In fact, in the second and third models, three or more reported ASB were significantly associated with increased odds of attempting suicide {(aOR 1.81 (1.11–2.96)}.

**Table 2:** Odds Ratios from multinomial logistic regression analyses of suicide attempts.

	Unadjusted Model	Model 1^a^	Model 2^b^	Model 3^c^
ASB	OR (95%CI)	aOR (95%CI)	aOR (95%CI)	aOR (95%CI)
*None Reported (Reference)*	1.00	1.00	1.00	1.00
*One Reported*	**1.30 (1.06-1.59)**	**1.34 (1.10-1.64)**	1.18 (0.94-1.48)	1.19 (0.96-1.47)
*Two Reported*	**1.52 (1.20-1.92)**	**1.58 (1.26-1.99)**	1.28 (0.97-1.68)	1.29 (1.00-1.68)
*Three or More Reported*	**2.68 (1.68-4.28)**	**2.85 (1.82-4.47)**	**1.74 (1.05-2.89)**	**1.81 (1.11-2.96)**

*OR: Odds Ratio; aOR: adjusted Odds Ratio; 95%CI: 95% Confidence Interval.*

^a^ *Adjusted for age, sex, race/ethnicity, survey year.*

^b^ *Adjusted for age, sex, race/ethnicity, survey year, family income, major depressive episode over the past year with severe role impairment, spoke to a professional in the past year, received treatment/counseling or medications in the past year, past-year tobacco use, past-year alcohol use disorder, past-year cannabis use disorder, past-year drug use disorder excluding cannabis, on parole or probation over the past year, moved over the past twelve months.*

^c^ *Adjusted for age, sex, race/ethnicity, survey year, family income, major depressive episode over the past year with severe role impairment, received treatment/counseling or medications in the past year, past-year tobacco use, past-year alcohol use disorder, past-year drug use disorder excluding cannabis, moved over the past twelve months.*

*Significantly different from “None” in* ***Bold*** *(p < 0.05)*

## Discussion

Our study aimed to investigate factors that are associated with attempting suicide among adolescents with suicidal ideations and plans.

Our results show that those who attempted suicide in this subpopulation were more likely to be girls, from lower socioeconomic background, those who’ve had a major depressive episode with severe impairment and have received professional help for depression in the year preceding the assessment. In addition, tobacco use, substance and alcohol use disorders were all associated with suicide attempts. Finally, we found an incremental association between antisocial behavior and suicide attempts in this population of adolescents with suicidal ideations and plans.

In line with previous findings on predictors of suicide attempts among adolescents [20,21] our results show gender as a significant predictor among ideators with a plan, with higher rates among females than males. In general, research has shown that female adolescents have a higher life-time prevalence of suicide attempts [22,23]. Our findings also align with prior research indicating that major depressive disorder is strongly correlated with the likelihood of suicide attempts among adolescents [21,24]. More specifically, in 2006 Spann et al. suggested that depressive symptomatology characterized by hopelessness was significantly associated with attempts [25]. In addition, we found that adolescents who attempted suicide were more likely to have sought help recently. Similarly, studies have indicated elevated rates of contact between patients and healthcare professionals prior to suicidal attempts, both fatal and non-fatal [26].

In line with prior research [27,28], our findings indicate that substance use heightens the risk of suicide attempts in adolescents. A more recent meta-analysis further supports this association, showing that substance misuse—including alcohol, tobacco, cannabis, opioids, and stimulants—is significantly associated with increased suicide mortality, with particularly elevated risk observed among female adolescents. Indeed, substance use contributes to suicide attempts via multiple pathways. Acute pharmacological effects of drug intoxication may impair judgment, lower inhibitions, worsen impulse control, and affect specific neurotransmitter systems, which increase the likelihood of suicide attempts [29]. Substance use can also trigger suicidal behavior by compromising developmental tasks [27] and triggering changes in behavioral, affective, and cognitive processes leading to dysregulation of aggression and impulsivity [30].

Along the same lines, we found that violent behavior, particularly involvement in fights and attacking with intent to harm, increased the risk of suicide among adolescents with ideations and a plan. This finding aligns with John Mann’s stress-diathesis model, which underscores the significance of aggression in suicidal tendencies, particularly how disinhibition toward certain behaviors can lead to harm to oneself or others. [31]. Across numerous studies involving high-risk individuals, trait aggression has consistently emerged as a predictive factor for future suicide. A recent meta-analysis further supports this association, showing that trait aggression and impulsivity are significantly linked to suicidal behavior [32]. Additionally, research conducted in school settings indicates that adolescents contemplating suicide tend to display elevated levels of aggression, highlighting its relevance as an indicator of suicidal vulnerability [33]. Even though this understanding of increased impulsivity among suicide attempters has been demonstrated in the literature, whether through substance use or violent tendencies, ours is the first study to highlight it in a sample of adolescents with suicidal ideations and plan.

The association between antisocial behavior and suicide has been reported in previous studies. In a national survey of juvenile correctional facilities from 2000 to 2014, for instance, suicide rates were consistently 2–3 times higher for youths in custody than for those in the general population [34]. Another study found that antisocial behavior and juvenile delinquency predicted an increased risk for suicidal ideations and behaviors [16]. Our findings further supported the aforementioned association, but within a high-risk population. This association was incremental with a particularly pronounced increase in the risk of suicide attempts when three or more antisocial behaviors were present.

Some studies have investigated individual or specific antisocial behaviors. For instance, one study found that driving while intoxicated was an independent risk factor for suicidal ideations and attempts [35]. Our study aligns with this finding, revealing that having a Driving Under the Influence (DUI) charge is associated with elevated rates of suicide attempts (p = 0.005).

Moreover, our study revealed a significant association between being arrested, on parole, or on probation and an elevated risk of suicide. Existing literature confirms that contact with the justice system, at any juncture, constitutes a risk factor for suicide among delinquent youth in both community and incarcerated settings. Among incarcerated youths, prevalence rates of recent suicide attempts varied from 3.0% to 8.5%, with lifetime attempts ranging from 11.0% to 29.4% [36]. Additionally, in a sample of young African-American adults, lifetime arrests were associated with lifetime suicidal ideation [37]. Conversely, we found no association between possessing a firearm and suicide attempts. A reason for this could be that firearms are fatal means of suicide and are typically associated with a higher risk of completed suicide [38]. The sample in our study only included survivors of suicide attempts.

While it is important to note that our findings do not imply a causal relationship, gaining a nuanced understanding of the theories influencing the transition from planning to attempting suicide is crucial. In fact, recent theories on suicide align with the ‘ideation to action’ framework [17]. This framework suggests that the emergence of suicidal thoughts and the transition from thoughts to attempts are distinct processes with different risk factors. It also emphasizes the role of “suicide capability” in this transition [3] which is believed to grow by experiencing painful and provocative events, resulting in an increased ability to tolerate pain, fear, and death. One aspect of this theory is the association between substance use and the increased risk of suicide attempts demonstrated in our study. In fact, engaging in substance use may correlate with a reduced fear of death [39] due to its potentially fatal consequences. This would consequently increase an individual’s acquired capability of suicidal behavior [40]. Along the same lines, youths who engage in potentially harmful or “risk-taking” behaviors such as antisocial behaviors would be expected to have increased capabilities to attempt suicide especially in the context of suicidal ideations as they have a high ability to tolerate painful and fear-inducing events [41].

While our findings offer valuable insights into suicide predictors, they also come with several limitations. Firstly, the use of a self-report questionnaire complicates the determination of whether these adolescents have been convicted of crimes or placed in correctional facilities. It also exposes the study to recall bias and potential misreporting of certain information.

Secondly, the cross-sectional design of our study prevents the establishment of a clear time frame for the occurrence of suicide attempts. In other words, the temporality of the attempts cannot be established, nor whether the reported attempts were related to the reported plans. Moreover, it is important to note that, due to the cross-sectional nature of the analysis, the non-attempters remain a high-risk group, and a proportion may “convert” to becoming attempters in the future.

Additionally, the assessment of risk relied solely on two questions, thus hindering our ability to ascertain the accuracy of suicide risk assessment. While one question assessed lifetime suicide attempts and the other focused on suicidal ideation during the worst time period, this discrepancy introduces temporal ambiguity, making it unclear whether the plan and the attempt occurred during the same episode. Moreover, factors such as social context, premeditation, impulsivity, and the circumstances surrounding the suicide attempt itself were not evaluated. The dataset also lacked measures of childhood abuse and neglect—established risk factors for suicidal behavior—limiting our ability to control for these important confounders. While protective factors are also critical in understanding suicide risk, they were not available in the NSDUH dataset and thus could not be included in our analysis. Similarly, although other psychiatric conditions are relevant, the NSDUH provides limited diagnostic data; we focused on major depressive episodes as the most consistently assessed and available condition. Lastly, because the NSDUH excludes institutionalized youth (e.g., those who are hospitalized or incarcerated), the findings may not generalize to higher-risk populations who are overrepresented in these settings. Our study has several notable strengths. Firstly, we utilized a large, nationally representative sample, which enhances the generalizability of our findings. Secondly, in contrast to the majority of literature examining the correlation between antisocial behaviors and suicidality, our study involved a non-institutionalized sample. This approach allows for a more accurate representation of the effect of the antisocial behaviors themselves, rather than the impact of involvement in the justice system. Finally, to our knowledge, ours is the first study to investigate the predictors of the transition from suicidal ideations and planning to attempting.

## Conclusion

This study aimed to identify factors among adolescents associated with transitioning from suicidal ideations with a plan to suicide attempt. Our findings revealed significant associations between suicide attempts and gender, socioeconomic status, major depressive disorder, substance use disorders, and antisocial behaviors. Particularly noteworthy was the pronounced increase in suicide risk associated with engaging in three or more antisocial behaviors. These findings underscore the importance of addressing mental health issues, substance use, and antisocial behaviors in suicide prevention efforts targeted at at-risk adolescents. Moreover, our study highlights the need for comprehensive risk assessment protocols that consider both individual and contextual factors. In conclusion, while providing valuable clinical insights, future research should explore these associations further and develop interventions targeting modifiable risk factors to prevent suicide attempts among vulnerable adolescents.

## References

[1] Ilic M, Ilic I. Worldwide suicide mortality trends (2000-2019): a joinpoint regression analysis. World J Psychiatry. 2022;12(8):1044–60. doi: 10.5498/wjp.v12.i8.1044 36158305 PMC9476842

[2] Hedegaard H, Curtin SC, Warner M. Suicide rates in the United States continue to increase. NCHS Data Brief. 2018;(309):1–8. 30312151

[3] Mars B, Heron J, Klonsky ED, Moran P, O’Connor RC, Tilling K, et al. Predictors of future suicide attempt among adolescents with suicidal thoughts or non-suicidal self-harm: a population-based birth cohort study. Lancet Psychiatry. 2019;6(4):327–37. doi: 10.1016/S2215-0366(19)30030-6 30879972 PMC6494973

[4] Pfledderer CD, Burns RD, Brusseau TA. School environment, physical activity, and sleep as predictors of suicidal ideation in adolescents: evidence from a national survey. J Adolesc. 2019;74:83–90. doi: 10.1016/j.adolescence.2019.05.008 31176240

[5] Posner K, Oquendo MA, Gould M, Stanley B, Davies M. Columbia classification algorithm of suicide assessment (C-CASA): classification of suicidal events in the FDA’s pediatric suicidal risk analysis of antidepressants. Am J Psychiatry. 2007;164(7):1035–43. doi: 10.1176/ajp.2007.164.7.1035 17606655 PMC3804920

[6] Harmer B, Lee S, Duong T vi H, Saadabadi A. Suicidal ideation. In: StatPearls. Treasure Island (FL): StatPearls Publishing; 2021. 33351435

[7] Nock MK, Green JG, Hwang I, McLaughlin KA, Sampson NA, Zaslavsky AM, et al. Prevalence, correlates, and treatment of lifetime suicidal behavior among adolescents: results from the National Comorbidity Survey Replication Adolescent Supplement. JAMA Psychiatry. 2013;70(3):300–10. doi: 10.1001/2013.jamapsychiatry.55 23303463 PMC3886236

[8] Geoffroy MC, Arseneault L, Girard A, Ouellet-Morin I, Power C. Association of childhood bullying victimisation with suicide deaths: findings from a 50-year nationwide cohort study. Psychol Med. 2023;53(9):4152–9. doi: 10.1017/S0033291722000836 35388770 PMC10317807

[9] Miché M, Hofer PD, Voss C, Meyer AH, Gloster AT, Beesdo-Baum K, et al. Mental disorders and the risk for the subsequent first suicide attempt: results of a community study on adolescents and young adults. Eur Child Adolesc Psychiatry. 2018;27(7):839–48. doi: 10.1007/s00787-017-1060-5 29027588 PMC6013520

[10] Fegg M, Kraus S, Graw M, Bausewein C. Physical compared to mental diseases as reasons for committing suicide: a retrospective study. BMC Palliat Care. 2016;15:14. doi: 10.1186/s12904-016-0088-5 26860949 PMC4746811

[11] Mars B, Heron J, Klonsky ED, Moran P, O’Connor RC, Tilling K, et al. What distinguishes adolescents with suicidal thoughts from those who have attempted suicide? A population-based birth cohort study. J Child Psychol Psychiatry. 2019;60(1):91–9. doi: 10.1111/jcpp.12878 29492978 PMC6334515

[12] May AM, Czyz EK, West BT. Differentiating adolescent suicide attempters and ideators: a classification tree analysis of risk behaviors. J Adolesc Health. 2020;67(6):837–50. 32576482 10.1016/j.jadohealth.2020.04.018 PMC7959206

[13] Björkenstam E, Björkenstam C, Vinnerljung B, Hallqvist J, Ljung R. Juvenile delinquency, social background and suicide--a Swedish national cohort study of 992,881 young adults. Int J Epidemiol. 2011;40(6):1585–92. doi: 10.1093/ije/dyr127 22158668

[14] Agerbo E, Nordentoft M, Mortensen PB. Familial, psychiatric, and socioeconomic risk factors for suicide in young people: nested case-control study. BMJ. 2002;325(7355):74. doi: 10.1136/bmj.325.7355.74 12114236 PMC117126

[15] Klonsky ED, May AM. Differentiating suicide attempters from suicide ideators: a critical frontier for suicidology research. Suicide Life Threat Behav. 2014;44(1):1–5. doi: 10.1111/sltb.12068 24313594

[16] Thompson MP, Ho C, Chua C, Kingree JB. Prospective associations between delinquency and suicidal behaviors in a nationally representative sample. J Adolesc Health. 2007;40(3):232–7. 17321423 10.1016/j.jadohealth.2006.10.016

[17] Klonsky ED, Qiu T, Saffer BY. Recent advances in differentiating suicide attempters from suicide ideators. Curr Opin Psychiatry. 2017;30(1):15–20. doi: 10.1097/YCO.0000000000000294 27798483

[18] Voss C, Ollmann TM, Miché M, Venz J, Hoyer J, Pieper L, et al. Prevalence, onset, and course of suicidal behavior among adolescents and young adults in Germany. JAMA Netw Open. 2019;2(10):e1914386. doi: 10.1001/jamanetworkopen.2019.14386 31664450 PMC6824228

[19] National Survey on Drug Use and Health (NSDUH). 2025 Jul 1. In: Substance Abuse and Mental Health Services Administration (SAMHSA). Available from: https://www.samhsa.gov/data/data-we-collect/nsduh-national-survey-drug-use-and-health 27748105

[20] Esang M, Ahmed S. A closer look at substance use and suicide. Am J Psychiatry Resid J. 2018;13(6):6–8.

[21] Haghish EF, Nes RB, Obaidi M, Qin P, Stänicke LI, Bekkhus M. Unveiling adolescent suicidality: holistic analysis of protective and risk factors using multiple machine learning algorithms. J Youth Adolesc. 2024;53(3):507–25. 37982927 10.1007/s10964-023-01892-6 PMC10838236

[22] Miranda-Mendizabal A, Castellví P, Parés-Badell O, Alayo I, Almenara J, Alonso I. Gender differences in suicidal behavior in adolescents and young adults: systematic review and meta-analysis of longitudinal studies. Int J Public Health. 2019;64(2):265–83. 30635683 10.1007/s00038-018-1196-1 PMC6439147

[23] Veloso-Besio C, Cuadra-Peralta A, Gallardo-Peralta L, Cuadra-Fernandez P, Quiroz PT, Troncoso NV. The prevalence of suicide attempt and suicidal ideation and its relationship with aggression and bullying in Chilean adolescents. Front Psychol. 2023;14:1133916. doi: 10.3389/fpsyg.2023.1133916 37275702 PMC10234288

[24] Carballo JJ, Llorente C, Kehrmann L, Flamarique I, Zuddas A, Purper-Ouakil D, et al. Psychosocial risk factors for suicidality in children and adolescents. Eur Child Adolesc Psychiatry. 2020;29(6):759–76. doi: 10.1007/s00787-018-01270-9 30684089 PMC7305074

[25] Spann M, Molock SD, Barksdale C, Matlin S, Puri R. Suicide and African American teenagers: risk factors and coping mechanisms. Suicide Life Threat Behav. 2006;36(5):553–68. doi: 10.1521/suli.2006.36.5.553 17087634

[26] Rothes I, Henriques M. Health professionals facing suicidal patients: what are their clinical practices? Int J Environ Res Public Health. 2018 Jun;15(6):1210. 29890677 10.3390/ijerph15061210 PMC6024946

[27] Wang P-W, Yen C-F. Adolescent substance use behavior and suicidal behavior for boys and girls: a cross-sectional study by latent analysis approach. BMC Psychiatry. 2017;17(1):392. doi: 10.1186/s12888-017-1546-1 29216850 PMC5721537

[28] Wolitzky-Taylor KB, Castriotta N, Lenze EJ, Stanley MA, Craske MG. Anxiety disorders in older adults: a comprehensive review. Depress Anxiety. 2010;27(2):190–211. doi: 10.1002/da.20653 20099273

[29] Stanley B, Michel CA, Galfalvy HC, Keilp JG, Rizk MM, Richardson-Vejlgaard R. Suicidal subtypes, stress responsivity and impulsive aggression. Psychiatry Res. 2019;280:112486. 31376789 10.1016/j.psychres.2019.112486 PMC7027392

[30] Sharma L, Markon KE, Clark LA. Toward a theory of distinct types of “impulsive” behaviors: a meta-analysis of self-report and behavioral measures. Psychol Bull. 2014;140(2):374–408. doi: 10.1037/a0034418 24099400

[31] Mann JJ, Waternaux C, Haas GL, Malone KM. Toward a clinical model of suicidal behavior in psychiatric patients. Am J Psychiatry. 1999;156(2):181–9. doi: 10.1176/ajp.156.2.181 9989552

[32] Moore FR, Doughty H, Neumann T, McClelland H, Allott C, O’Connor RC. Impulsivity, aggression, and suicidality relationship in adults: a systematic review and meta-analysis. EClinicalMedicine. 2022;45:101307. doi: 10.1016/j.eclinm.2022.101307 35243273 PMC8860929

[33] Greening L, Stoppelbein L, Luebbe A, Fite PJ. Aggression and the risk for suicidal behaviors among children. Suicide Life Threat Behav. 2010;40(4):337–45. doi: 10.1521/suli.2010.40.4.337 20822360

[34] Ruch DA, Sheftall AH, Schlagbaum P, Fontanella CA, Campo JV, Bridge JA. Characteristics and precipitating circumstances of suicide among incarcerated youth. J Am Acad Child Adolesc Psychiatry. 2019;58(5):514-524.e1. doi: 10.1016/j.jaac.2018.07.911 30768395 PMC9721273

[35] Kim N, Lee J, Hahm BJ, Yang BR. Association of driving while intoxicated and suicide ideation and attempts in South Korea: a study in a nationally representative sample. Sci Rep. 2023;13:14199. 37648687 10.1038/s41598-023-40829-8 PMC10468494

[36] Stokes ML, McCoy KP, Abram KM, Byck GR, Teplin LA. Suicidal ideation and behavior in youth in the juvenile justice system: a review of the literature. J Correct Health Care. 2015;21(3):222–42. doi: 10.1177/1078345815587001 26084946 PMC5704936

[37] Ahuja M, Records K, Haeny AM, Gavares EM, Mamudu HM. The association between experiencing police arrest and suicide ideation among emerging young adults: does race matter?. Health Psychol Open. 2021;8(1). doi: 10.1177/20551029211026027 PMC8221685 34221440

[38] Spark TL, Wright-Kelly E, Ma M, James KA, Reid CE, Brooks-Russell A. Assessment of rural-urban and geospatial differences in perceived handgun access and reported suicidality among youth in Colorado. JAMA Netw Open. 2021;4(10):e2127816. doi: 10.1001/jamanetworkopen.2021.27816 34623407 PMC8501400

[39] Bayliss LT, Christensen S, Lamont-Mills A, du Plessis C. Suicide capability within the ideation-to-action framework: a systematic scoping review. PLoS One. 2022;17(10):e0276070. doi: 10.1371/journal.pone.0276070 36301944 PMC9612581

[40] Ammerman BA, Steinberg L, McCloskey MS. Risk-taking behavior and suicidality: the unique role of adolescent drug use. J Clin Child Adolesc Psychol. 2018;47(1):131–41. doi: 10.1080/15374416.2016.1220313 27732082 PMC6082017

[41] Sewell AA, Jefferson KA. Collateral damage: the health effects of invasive police encounters in New York City. J Urban Health. 2016;93 Suppl 1(Suppl 1):42–67. doi: 10.1007/s11524-015-0016-7 26780583 PMC4824697

